# A Robust Framework for Domain-Generalized Classification of Ovarian Cancer Histology Images

**DOI:** 10.3390/diagnostics15232954

**Published:** 2025-11-21

**Authors:** Awais Ahmed, Xiaoyang Zeng

**Affiliations:** 1 School of Computer Science, China West Normal University, Nanchong 637009, China; 2 School of Computer Science and Engineering, University of Electronic Science and Technology of China—UESTC, Chengdu 611731, China; 202011081605@std.uestc.edu.cn

**Keywords:** whole slide image, histology image analysis, downscaled patch sampling, multiple instance learning, ovarian cancer classification

## Abstract

**Background:** In computational pathology (CP) analysis, computational efficiency and precise classification outcomes are paramount for robust and scalable solutions. Despite recent advancements in deep-learning frameworks for Whole-Slide Images (WSIs), the heterogeneity of WSIs across different domains poses considerable challenges for developing models with robust generalization capabilities. This study presents the WSI-P2P (Whole-Slide Imaging–Patch to Prediction), aimed at addressing these challenges. **Methods:** WSI-P2P leverages downscaled patch sampling and Multiple-Instance Learning (MIL) with transfer learning to optimize resource usage while maintaining a competitive performance. Within WSI-P2P, we introduce the *K-TOP* MIL aggregator, a variant of the MIL attention-based aggregator, which selectively processes the most informative *K* instances. The framework features an online, adaptive feature extractor that fine-tunes pre-trained models in an end-to-end manner, addressing multi-centered dataset variability. **Results:** WSI-P2P achieves state-of-the-art accuracy, demonstrating superior domain adaptability and computational efficiency. WSI-P2P is validated by employing several dataset splits and down-sampled patch variations, illustrating its potential as a scalable and reliable tool in clinical settings and large-scale histological studies. The framework achieved a maximum score of 95.89% AUROC and a test accuracy of 77.67% without attention, further improving to approximately 100% AUROC and a test accuracy of 95.72% recorded with the *K-TOP* MIL aggregator. Further, for intra-domain generalization experiments, WSI-P2P recorded a consistent performance across domains, validating its domain generalization capabilities. The *K-TOP* MIL aggregator also demonstrated 2.3× computational efficiency as compared to base aggregators. **Conclusions:** The proposed framework outperforms traditional offline feature extraction methods, ensuring high discriminative ability even when exposed to data from diverse distributions. WSI-P2P demonstrates excellent performance between subtype classifications, positioning it as a reliable tool for large-scale histological studies.

## 1. Introduction

Computational pathology (CP) has emerged as a promising approach for digital pathology over the past decade, leveraging advanced algorithms and machine learning techniques to automate and enhance the analysis of histopathological data [1,2,3,4,5]. This innovative technique helps to augment pathological assessment diagnostic accuracy, speed, and efficiency, bridging the gap between traditional microscopy and modern digital technologies [6]. Given the profound impact of CP on advancing diagnostic precision and efficiency, its application extends far beyond traditional pathology boundaries, embracing a broader spectrum of histology applications [3,7]. Among these, the classification of ovarian cancer subtypes emerges as a particularly promising avenue [8,9,10,11,12].

Recent advances in artificial intelligence (AI) have demonstrated significant potential across gynecological oncology, from diagnostic pathology to surgical interventions [13]. The integration of AI in clinical workflows requires robust validation and standardization of protocols, as evidenced by successful deployments in gynecological surgery [14]. However, bridging the gap between computational innovation and clinical adoption necessitates careful evaluation of human–AI collaboration dynamics [14,15].

Ovarian cancer, which is characterized by its heterogeneity and various subtypes, each with distinct histological features, prognoses, and treatment responses, is becoming one of the most lethal gynecological malignancies [16,17,18,19,20,21]. Within Whole-Slide Images (WSI), histology image analysis plays a crucial role in diagnosing and understanding cancerous tissues, providing valuable insights for treatment decisions and prognostic assessments [22,23]. WSIs have revolutionized this domain recently, offering a digital framework for analyzing tissue samples [12,24,25,26,27]. WSIs capture tissue sections at microscopic resolution, resulting in images that span over billions of pixels [28], consisting of rich diagnostic information crucial for identifying cancer subtypes and predicting patient outcomes [29,30].

Machine learning and computer vision advancements have revolutionized histology image analysis in recent years, offering automated and scalable cancer diagnosis and classification solutions [24,31,32,33,34]. However, the computational demands of processing high-resolution histology images pose significant challenges, particularly in large-scale datasets. Processing high-resolution histology images presents major computational hurdles in numerous medical imaging applications, such as diagnosing and categorizing malignant tissues. In addition, automated analysis, which is made possible by machine learning and computer vision developments, frequently depends on abundant labeled data from the same domain. This reliance on specific data limits the ability of these methods to be applied to new datasets or imaging settings. The challenge is especially prominent in histology image analysis, since differences in staining processes, tissue preparation techniques, and imaging modalities can cause domain changes that impede the performance of models. Addressing these challenges requires innovative approaches to enhance computational efficiency without compromising classification accuracy.

While MIL provides a systematic and effective strategy for leveraging weak supervision [5,35,36], managing ambiguity and variability [37], and scaling to extensive datasets [38,39], processing high-resolution images still imposes substantial computational burdens [32,40]. To mitigate this, WSI-P2P utilizes downscaled patch sampling to reduce overall model training size as well as inference time; together, both make it robust and computationally efficient. Further, by employing *K-TOP*, a MIL aggregator, in addition to the attention score, it also reduces resource consumption and selects the most valuable patches for inference. Secondly, there is a need to develop models that can generalize effectively across diverse domains while utilizing bag-level labels. WSI-P2P addresses this challenge by ensembling the pretrained transferred knowledge. The proposed framework is evaluated on the UBC-OCEAN dataset [41]; the data is taken from Kaggle (https://www.kaggle.com/competitions/UBC-OCEAN/overview, accessed on 13 June 2025). Comparative experiments with SOTA work and submission scores at Kaggle suggest the potential for real-time deployment.

The main contributions of this work are as follows:

- **WSI-P2P Framework:** A Novel Integration of Downscaling and MIL
  – To the best of our knowledge, WSI-P2P is the first framework to combine diagnosis-aware downscaling with a *K-TOP* MIL aggregator.
  – This framework is designed to efficiently process WSIs for the domain-generalized classification of ovarian cancer histology images, addressing both computational efficiency and performance.
  – Further, we optimize the attention-based MIL aggregation method by integrating *K-TOP* instance selection, which selectively processes the most informative *K* number of instances, reducing computational costs compared to traditional WSI processing methods.
- **Multi-Task Analysis:** This work primarily focuses on the classification of ovarian cancer histology images, where accuracy and AUC metrics are compared, while the subtype classification task is also validated; it also demonstrated stable performance in terms of balanced accuracy in comparison to the latest works, with our *K-TOP* MIL aggregator.
- **Robust Domain Generalization:** Unlike traditional approaches, we leverage transfer learning within the MIL framework, where WSI-P2P demonstrated robust generalization capabilities across domains.
- **Competitive Classification Performance:** Our experimental results demonstrate WSI-P2P’s superiority in ovarian cancer intra-domain generalization and subtype classification, highlighting the potential for real-time clinical deployment.

The proposed framework may be considered when developing real-time applications that address several areas recommended by the World Health Organization (WHO) and the European Society of Gynecological Oncology (ESGO), including histotype differentiation, tumor grading, biomarker identification, prognostic assessment, treatment response prediction, and quality control diagnostics [42]. The objectives are to achieve minimal resource consumption and robust performance, as this study aims to optimize computational efficiency and improve performance in ovarian cancer classification.

The rest of the study is organized as follows: Section 3 presents a detailed overview of dataset preparation steps; further, Section 4 details the methodology of the proposed work. Section 5 discusses system implementation details, then Section 6 presents the experimental evaluations. Further study limitations and future work, along with the conclusions, are discussed in Section 7 and Section 8, respectively.

## 2. Related Work

Multiple instance learning (MIL) has emerged as a key tool in the emerging field of computational pathology. This section briefly describes the existing literature work covering MIL, domain generalization, and ovarian cancer classification.

### 2.1. Multiple Instance Learning

With the recent advancement, MIL has emerged as a best-fit approach for computational pathology and its challenges. Given the computational costs of processing high-resolution images containing billions of pixels, obtaining pixel-level annotations in the medical field is challenging, leading to a scarcity of annotated data. A compelling strategy involves partitioning a WSI into smaller patches, subsequently treating each patch/tile as a “bag” with an associated single label, as proposed by [43] in 2017. This concept has garnered significant interest in computational histopathology, where individual patches may correlate to cellular structures indicative of cancerous transformations.

Diverse MIL methodologies have been applied to histopathological data. Examples include Gaussian processes, as explored by [44,45] in 2014 and 2016, respectively, and a combination of neural networks with an Expectation-Maximization (EM) algorithm for classifying instances was proposed by study [46] in 2016. Following foundational work by [46], various works have been conducted. One recent work proposed by [47] utilizes EM for domain adaptation for a perineural invasion and nerve extraction task in whole-slide digital pathology images. Furthermore, MIL has also found applications beyond histopathology, such as in mammographic nodule classification [48] and in detecting cells within microscopy images [49].

Accurately identifying these subtypes through CP enhances diagnostic accuracy and tailors treatment strategies to individual patient needs. This significantly improves clinical outcomes and facilitates more precise and comprehensive interpretations of cancerous tissues.

### 2.2. Medical Domain Generalization

Domain generalization techniques have arisen as a viable method to create robust models that can effectively generalize across various datasets and imaging settings [50,51,52,53]. These techniques seek to enhance models’ resilience and adaptability to unfamiliar data by acquiring knowledge that remains unchanged despite differences particular to different domains [54]. By incorporating its concepts into histological image analysis, the scalability and effectiveness of automated diagnosis systems can be improved. This allows them to consistently perform well across various medical institutions and imaging setups [55,56].

#### MIL-Based Domain Generalization

While standard MIL-based methods have received significant attention and have also demonstrated remarkable performances in computational pathology, they often degrade significantly under domain shift scenarios caused by inter-institutional variations in staining protocols, scanning techniques, and tissue preparation approaches, etc. Recent research has begun addressing these critical challenges by leveraging advanced deep learning techniques; MIL architectures among the techniques used for improving domain generalization.

Several innovative approaches have emerged [50,57,58], yet they often struggle with computational efficiency due to the complex interplay between instance-level variations and bag-level labels. To tackle these issues, researchers have proposed several approaches, including the integration of attention mechanisms to emphasize pertinent features within bags, thereby improving the model’s interpretability and performance [5,33,59]. Further, these methods are briefly discussed in our SOTA Analysis (Section 6.5). Despite these advancements, there remains a need for more efficient algorithms that can balance the computational demands of training with the necessity for robust performance in diverse clinical settings. Our study contributes by introducing the MIL-based *K-TOP* aggregator, which seeks to optimize performance while addressing computational challenges inherent in MIL-based domain generalization.

### 2.3. Ovarian Subtype Classification

When it comes to malignant tumors, ovarian cancer still has one of the worst survival rates. Ovarian cancer histopathology subtyping plays a critical role in determining patient treatment protocols, with five major subtypes exhibiting distinct molecular profiles and clinical outcomes. While WSI has digitized pathological workflows, computational analysis remains challenged by fundamental limitations [5,12,60,61] such as

- Gigapixel-scale regulations requiring intensive processing.
- Heterogeneity across medical centers in staining protocols and scanner systems.
- Weak slide-level labels that lack precise tumor region annotations.

Recent attention on ovarian cancer [8,32,33,41,62] is highlighting the demand for a robust solution to avoid losses of human lives as reported by [63], without improved methods of prevention or control, it is projected that ovarian cancer will cause damage to approximately eight million lives from 2022 to 2050 [64]. The current study addresses the aforementioned challenges with its innovative methodology.

## 3. Data Collection and Preprocessing

The study introduces a diagnosis-aware down-scaling protocol, Whole-Slide Image (WSI) processing, leveraging the UBC-Ovarian Cancer Challenge dataset [41]. The approach addresses two critical challenges in computational pathology: (i) preserving histomorphological features at reduced resolution and (ii) enabling efficient large-scale analysis without compromising diagnostic validity.

**Figure 1:**
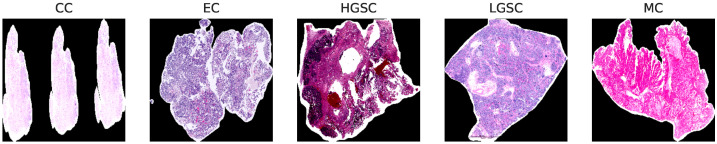
A representative histology sample of five distinct subtypes of ovarian cancer. Reprinted from Zeng et al. [57].

The original dataset consists of WSI from diverse medical centers, introducing variability and making it a valuable resource for evaluating generalization in computational pathology. Variability occurs due to differences in staining protocols, scanner systems, and tissue preparation. Overall, data is categorized into five distinct classes representing different tissue types: Clear Cell Carcinoma (CC), Mucinous Carcinoma (MC), Low-Grade Serous Carcinoma (LGSC), High-Grade Serous Carcinoma (HGSC), and Endometrioid Carcinoma (EC); we prepared the representative sample of the subtyping as shown in Figure 1.

**Data preprocessing:** The WSIs represent a vast and high-dimensional data space due to their large size and high-resolution details. To reduce the computational complexity, the study adopted downscaled patch sampling techniques. For each WSI, *N* random slices or tiles were selected, where *N* was set to 50 for this study. The tile selections were carefully performed to identify the matter within each WSI, allowing the subsequent analysis to focus on areas of diagnostic significance and discard the irrelevant background. Each WSI tile is recorded at a 512×512 size. WSIs were handled by the PIL pipeline, ensuring effective handling of their large file sizes and complex data structures. For WSI cropping, various techniques were observed, including but not limited to (i) signal smoothing, (ii) peak detection, (iii) image cropping, and (iv) signal scaling.

Furthermore, we have employed efficient preprocessing techniques to enhance data quality while minimizing resource consumption. This includes techniques such as noise reduction, data normalization, and feature extraction, which are computationally lightweight yet effective in improving the accuracy of the models. During the processing of WSI, we faced challenges, including a decompression bomb error. Several techniques were considered to mitigate this issue: decreasing the image resolution, implementing lazy loading, and resizing images. Furthermore, we have employed efficient preprocessing techniques to enhance data quality while minimizing resource consumption. This includes techniques such as noise reduction, data normalization, and feature extraction, which are computationally lightweight yet effective at improving the accuracy of the models. During the processing of WSI, we faced challenges, including a decompression bomb error. Several techniques were considered to mitigate this issue: decreasing the image resolution, implementing lazy loading, and resizing images. We also filter and validate the image size and utilize external libraries, setting the maximum pixel limit to none.

Finally, after preprocessing WSIs to collect small patches, we defined the data nature as one-vs.-50. Initially, it was one vs. one, termed as one-vs.-1 (one whole WSI as a single image); later, one WSI was divided into 50 small patches or tiles, as illustrated in Table 1. Now, the data is ready to be incorporated into our methodology for training, validation, and further investigation. Further details of downscaling are discussed in Section 4.

### Dataset Statistics

It is essential to provide a brief overview of the statistics from the original dataset following its official release.

**Figure 2:**
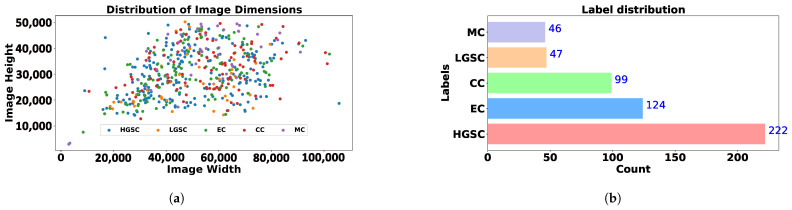
Data Statistics. (**a**) Scatter Plot; (**b**) Class Imbalance. Plot showing imbalance and scatter representation of employed dataset.

Initially, we present Figure 1, which emphasizes the need for preprocessing, as a significant portion of WSI histology images consists of multiple traces of the same slices. Then, we illustrate statistics in Table 2 further, highlighting the WSI billion pixels in Figure 2a. Finally, Figure 2b illustrates the dataset’s inherent imbalance. Although this study only presents the methodology for efficient preprocessing, the statistical information it provides is helpful for future researchers planning to use this dataset. Table 2 would benefit researchers planning to adopt this dataset for OOD-related tasks, and Figure 2b would help those who plan to investigate subtype imbalance classification problems.

## 4. Methodology

The study is inspired by the multiple-winning solutions to the ovarian cancer challenge, which have demonstrated the effectiveness of preprocessing techniques and the significant computational resources required. However, we recognize the need to address several challenges associated with resource reduction while maintaining a competitive performance. Further, this study is the first to have such a detailed investigation and propose a naive solution, WSI-P2P, that can potentially be employed as a healthcare industrial application where efficiently dealing with billions of pixels from WSI is necessary. The image size can be observed from the presented scatter plot as shown in Figure 2a, where both axes are evident in pixel size.

The approach consumes 4 times fewer resources than the first place-winning solution, which employed 200 random tiles for feature extraction and classification. This selection created a set of “N MIL Bags,” each containing a subset of slices representing different regions of the WSI. The images are organized into “bags,” each containing a fixed set of N images corresponding to a specific tissue type. This structure ensures the MIL framework, where the bag is the fundamental unit for learning and prediction. Further, we define a custom dataset class, CustomMILDataset, to handle the loading and transformation of the images. This class inherits PyTorch’s Dataset class, leveraging its inherent functionalities while customizing the getitem method to return a stack of transformed images (a bag) and a single label for that bag.

After data collection, several preprocessing and data augmentation steps were performed. Each image tile was resized to a uniform dimension of 224×224 pixels to ensure consistency. The preprocessing pipeline included several augmentation techniques to enhance the dataset’s diversity and robustness, such as random rotations, flips, color jittering, and Gaussian blurring. Depending on the requirements, the magnification of the images could be enhanced to levels such as 10×, 20×, 40×, etc., to capture details relevant to the specific diagnostic task. Several neural network pre-trained models served as feature extractors, transforming the raw image data into a high-dimensional feature space. The extracted features from each tile within a bag were then processed using an MIL approach. Each bag was associated with a label that applies to the collective set of instances (tiles) it contains rather than individual instance labels. Instances within a bag were considered for their potential to be positive (indicative of the label) or negative. The MIL approach utilized the correlation information between instances within a bag to infer the bag-level label. Not all patches (tiles) accurately inherit the WSI-level annotations due to the tissue’s heterogeneity and diverse morphological features within a single slide.

The following equations provide an overview of the methodological steps involved in preparing the WSI-P2P framework.

**Downscaled patch sampling:** Let $X_{i}$ denote the set of instances (tiles) in the *i*th bag, where each instance is a downscaled patch from a WSI. Our downscaling function is defined as follows:

(1) $$X_{i,j}=D\left(P_{i,j}\right)$$

where $P_{i,j}$ is the *j*th patch in the *i*th bag before downscaling, *D* is the downscaling operation, and $X_{i,j}$ is the downscaled patch, as shown in the diagram’s preprocessing box.

*Example calculation:* Let $N_{h}$ be the number of tiles along the horizontal axis, and $N_{v}$ be the number of tiles along the vertical axis.

The image’s dimensions are given as W×H, and the dimensions of each tile are R_W×R_H.

For example, for a WSI with a dimension of 16,000 × 16,000, the possible number of tiles along each axis is as follows:   

$$N_{h}=\left\lfloor \frac{16,000}{512}\right\rfloor =31$$

$$N_{v}=\left\lfloor \frac{16,000}{512}\right\rfloor =31$$

The total number of 512×512 would be the product of each axis:

$$\mathrm{Total}\;\mathrm{number}\;\mathrm{of}\;512\times 512\;\mathrm{tiles}=N_{h}\times N_{v}=31\times 31=961$$

In conclusion, a maximum of 961 non-overlapping 512×512 images can be generated.

**Adaptive patch extraction:** WSIs were partitioned into overlapping tiles (512×512 px at 20× magnification) with a 50-pixel stride to ensure tissue continuity. Black or empty tiles were filtered via brightness thresholding (mean intensity <25/255).

**Controlled resolution reduction:** Retained tiles were downscaled to 256×256 px using Lanczos resampling, achieving 4× memory reduction (e.g., 2.25 MB is reduced to 0.56 MB per tile), further comparative analysis between other recent works on the same UBC-OCEAN dataset is summarized in tabular form in Section 6.5, where we demonstrated WSI-P2P’s applicability.

**Feature extraction via transfer learning:** Below, we mathematically represent the feature extraction using the pre-trained model:

(2) $$F_{i,j}=M\left(X_{i,j};\Theta \right)$$

where $F_{i,j}$ denotes the feature vector extracted from the *j*th instance in the *i*th bag, *M* represents the feature extractor model applied to the instance $X_{i,j}$, and Θ denotes the parameters of the model.

**MIL aggregation function.** Given a bag $B_{i}=\left\{x_{i,1},\ldots ,x_{i,n}\right\}$ with instance features $F_{i,j}=f_{\theta }\left(x_{i,j}\right)$, the bag-level representation $B_{i}$ is computed as follows:

(3) $$B_{i}=A\left({\left\{F_{i,j}\right\}}_{j=1}^{n}\right),$$

where A is an aggregation operator.

We evaluate:

- *Standard aggregators*: Mean ($A_{\mathrm{mean}}$) and max ($A_{\max}$) pooling.
- *Attention pooling* ($A_{\mathrm{attn}}$): Learns instance weights $\alpha_{j}$ via a neural network.
- *K-TOP Score Pooling* ($A_{K\text{-}\mathrm{top}}$): Averages features from the top-*K* instances ranked by a learned scorer $s(F_{i,j})$.

Our proposed *K-TOP* aggregation mitigates noise by focusing on discriminative instances while preserving gradient flow.

Here, we hypothesize that the *K-TOP* feature works because in histopathology, only K number of tiles may contain tumor regions; averaging over all tiles dilutes signals. As compared to standard pooling (max, mean), *K-TOP* is approximated as a smoother alternative.

**Classification:** The final classification decision for a bag is modeled as follows:

(4) $$y_{i}=C\left(B_{i};\Phi \right)$$

where $y_{i}$ is the predicted label for the *i*th bag, *C* represents the classifier (e.g., a linear layer followed by a softmax operation in the case of multi-class classification) and Φ denotes the parameters of the classifier.

**Loss Function:** For a dataset with *N* number of bags, the loss function optimizing the parameters of the classifier is defined as follows:

(5) $$L\left(\Phi \right)=\frac{1}{N}\sum_{i=1}^{N}l\left(y_{i},{\hat{y}}_{i}\right)$$

where *l* is a loss function (e.g., cross-entropy) comparing the predicted label $y_{i}$ with the true label ${\hat{y}}_{i}$ of the *i*th bag.

### 4.1. MIL Aggregators

- Max pooling: Overfit to a single dominant tile (ignoring supportive evidence).
  (6) $$A_{\max}\left(\cdot \right)=\max_{j=1\ldots n}F_{i,j}\;\left(\mathrm{element}\text{-}\mathrm{wise}\right)$$
- Mean pooling: Averages signals with irrelevant/normal tiles (diluting discriminative features).
  (7) $$A_{\mathrm{mean}}\left(\cdot \right)=\frac{1}{n}\sum_{j=1}^{n}F_{i,j}$$
- Attention-based: Learn instance weights via $\alpha_{j}$ a neural network.
  (8) $$A_{\mathrm{attn}}\left(\cdot \right)=\sum_{j=1}^{n}\alpha_{j}F_{i,j},\;\alpha_{j}=\mathrm{softmax}(w^{⊤}\tanh(VF_{i,j})),$$
  where $w\in R^{m}$, $V\in R^{m\times d}$ are learnable parameters.
- The proposed attention-based K-TOP averages features from the top-*K* instances ranked by learned scorer $s(F_{i,j})$.
  (9) $$K\text{-}\mathrm{top}=\mathrm{Top}\text{-}K(F_{i},s\left(\cdot \right))$$

### 4.2. MIL Transfer Learning

The MIL architecture utilizes feature extractors such as ResNet50, ResNet18, and Vision Transformer ViT (where pre-trained = T/F). The Linear Neural Network (fully connected network (FCN)) is employed as a classifier layer. Further, the final fully connected layer of the feature extractor model is replaced with an identity layer to pass the extracted features directly to a custom classifier. The classifier consists of a linear layer with an output dimension equal to the number of classes, in this case, five. In the MIL context, the forward pass involves reshaping the input to process individual images through the feature extractor model and then aggregating the features within each bag using a max-pooling operation, whereas several other operations may be considered. With this operation, WSI-P2P selects the most prominent features across the instances in a bag, assuming that the most relevant features indicate the bag’s label.

For the classification task, the methodology employed in this study involves two distinct MIL approaches (with and without the attention approach), each utilizing different attention mechanisms. In the first approach, MIL Pooling (Max) is utilized without attention, where the maximum prediction score among instances within each bag is selected as the bag-level prediction. This method provides a straightforward aggregation strategy, leveraging the highest-scoring instance within each bag for classification. In contrast, the second approach incorporates attention by employing MIL Top *K* instances and pooling. Here, attention mechanisms are applied to identify and emphasize the most informative instances within each bag, determined by their relevance to the classification task. Subsequently, these top K instances are pooled together, potentially providing a more nuanced representation of the bag’s content. By integrating attention mechanisms into the MIL framework, this approach aims to enhance classification performance by focusing on the most discriminative instances while effectively aggregating their contributions at the bag level.

The attention score is defined as follows:

(10) $$\mathrm{Attention}\;\mathrm{Score}\left(x\right)=\mathrm{softmax}\left(\mathrm{Linear}\left(\mathrm{Tanh}\left(\mathrm{Linear}(x)\right)\right)\right)$$

**Figure 3:**
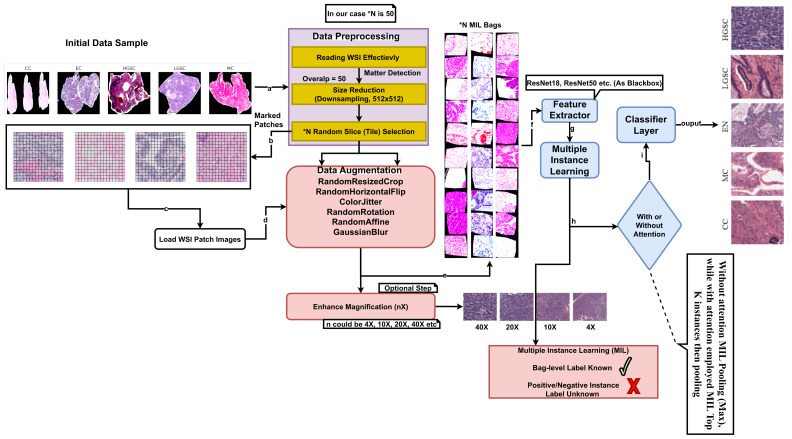
The WSI-P2P architectural framework. This diagram illustrates the proposed methodology, showcasing the workflow from down-scaled patch sampling to the final classification.

In conclusion, after downsizing large histology WSI images into small patches, the dataset was trained on pretrained models with further fine-tuning to achieve transfer learning, and various MIL aggregator comparisons were conducted. The methodology described above is briefly presented in an architectural diagram as depicted in Figure 3, with each step labeled for easy comprehension, and further high-level code steps are presented in Algorithm 1.
**Algorithm 1:** A High-level transfer Learning-based MIL algorithm for WSI-P2P
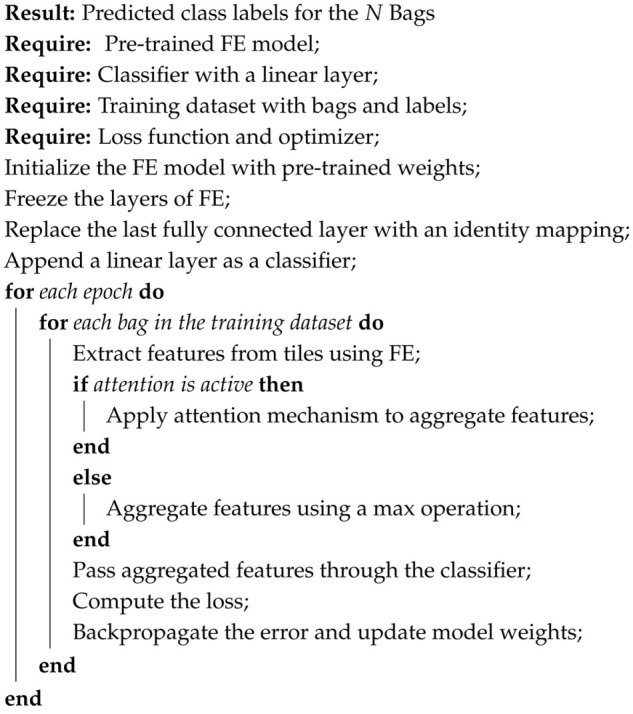


## 5. Implementation Details

### 5.1. System Parameters

For the experiments, an NVIDIA TITAN RTX GPU, equipped with driver version 470.161.03 and CUDA version 11.4, is utilized for computational operations. Additionally, the proposed work is implemented using Python 3.11 with PyTorch 1.13.0, a widely used deep-learning framework.

### 5.2. Experimental Parameters

Table 3 lists the comprehensive experimental configurations and hyperparameters employed in our proposed framework. These parameters were finalized through various iterations aimed at optimizing both computational efficiency and framework performance.

### 5.3. Baseline Models

In this study, we employ various variants of the ResNet model as baseline models, utilizing pre-trained weights to facilitate transfer learning for the classification of ovarian cancer histology images. Specifically, we employed ResNet18 and ResNet50, both initialized with pre-trained weights and later fine-tuned on our specific dataset to adapt their learned features to the unique characteristics of histological images.

### 5.4. Performance Metrics

We employ a comprehensive evaluation protocol assessing both classification performance and clinical reliability, utilizing the following set of equations.

(11a)Accuracy=TP+TNTP+TN+FP+FN(11b)Precision=TPTP+FP(11c)Recall(TPRate)=TPTP+FN(11d)Specificity(TNRate)=TNTN+FP(11e)F1=2×Precision×RecallPrecision+Recall(11f)BalancedAccuracy=TPR+TNR2(11g)Cohen’sKappa=Po−Pe1−Pe

## 6. Experiments and Discussion

This section provides a detailed description of the experimental setting used in this study to evaluate the performance and efficiency of the proposed WSI-P2P architecture. Furthermore, this study aims to verify the framework’s ability to classify ovarian cancer histology images in a domain-generalized manner. Additionally, this study seeks to evaluate its computational efficiency under various scenarios. Using various WSIs from multi-center studies, we investigate the effects of varying MIL bag sizes on the model’s learning dynamics and overall accuracy. Further model calibration is conducted. Additionally, emphasis is given to comparisons of MIL aggregators. The remaining experiments are performed on a curated dataset from the UBC-OCEAN dataset, as discussed in Section 3. A thorough comparative analysis with state-of-the-art (SOTA) methods is conducted to understand the nuances of our approach, thereby highlighting its strengths and potential areas for further improvement. In summary, the reported results were evaluated against multiple runs, and statistical significance was measured with a *p*-value below the accepted threshold of 0.001.

### 6.1. Comparative Analysis

To investigate the nuanced behavior of the proposed WSI-P2P framework while optimizing resource utilization, the variation in epoch sizes was purposefully determined according to the size of the MIL bags employed in our experiments. Concerning experiments involving an MBS of 10, we examined the model’s performance throughout 50 epochs. On the contrary, we restricted the training epochs to 25 epochs for models employing a MIL bag size of 25 and an additional time constraint of 10 epochs on models utilizing an MBS of 50. By employing this approach, we could evaluate the efficacy and flexibility of the framework at various MIL bags while incurring minimal computational burden. By conducting a thorough set of experiments, which involve comparing our approach with established benchmarks and baselines, we aim to highlight the proposed method’s benefits in effectively handling the inherent complexities of computational pathology.

**Figure 4:**
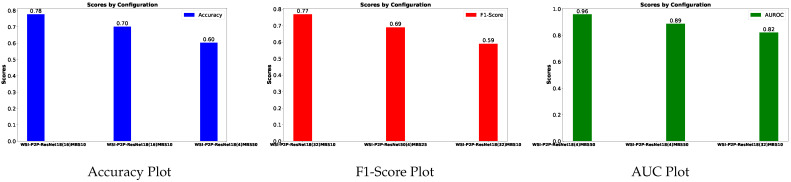
Best, average, and worst model performer of WSI-P2P with different configurations.

**Figure 5:**
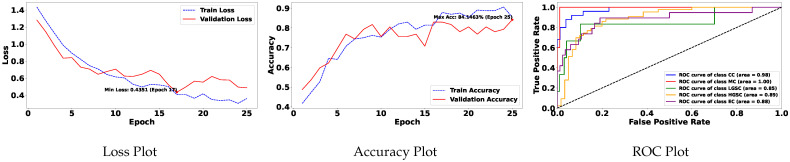
Training and validation loss, accuracy, and AUROC plots.

**Figure 6:**
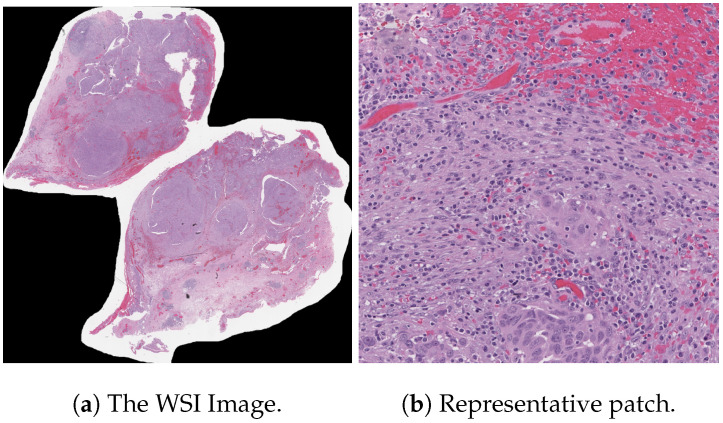
The subplots are (**a**) a 13364-HGSC Original H&E stained WSI image and (**b**) a version resized to 512×512 (one of the tiles). The Model-generated heatmaps for (**b**) are shown in (Figure 7).

**Figure 7:**
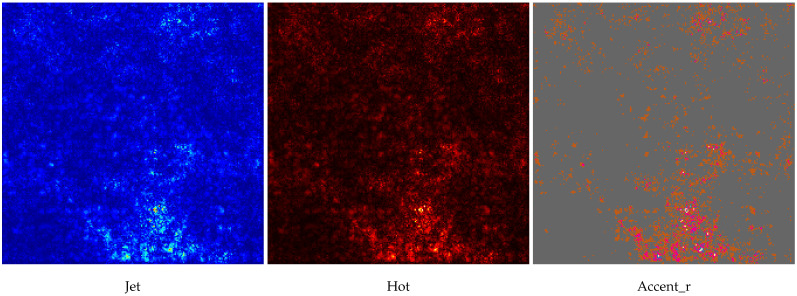
Heatmaps for the above-presented WSI in Figure 6. The interpretation for this heatmap plot is briefly discussed in the Appendix A.

Table 4 is prepared to illustrate the WSI-P2P model’s impact of variation of epoch size, then analyzing the impact with batch size, to further validate how the dataset split helps the model to learn effectively or helps to increase the model’s learning process. Further, we prepared a bar chart visualization to summarize and illustrate the top, average, and worst performers for accuracy, F1-score, and area under the curve. The bar chart in Figure 4 compares WSI-P2P-ResNet18 and WSI-P2P-ResNet50 feature extractor models with different batch sizes and data splits. These models were assessed using accuracy, F1-score, and AUROC. This comparative investigation shows that model selection and setup are crucial to the classification performance measures. Table 5 demonstrates the top 10 scored experiments from Table 4, while Figure 5 depicts the loss, accuracy, and roc curve trend over the epochs for the best-case scenario of Table 5. It records the maximum accuracy of training and validation while it also presents minimum loss values for training and validation of WSI-P2P with various network settings. For reference to our model results, we present an original WSI as shown in Figure 6a, while Figure 6b shows one of the representative patches. Overall, Figure 6 and Figure 7 depicts the model’s effectiveness and robustness towards the model’s explainability, which are further discussed in Appendix A.

#### Impact of Attention Mechanism and Top K Score

This section investigates the impact of the attention mechanism and top *K* score on the proposed study’s performance. Initially, without incorporating attention mechanisms, the proposed model attained a maximum AUROC (Area Under the Receiver Operating Characteristic curve) score of 95.89% and a test accuracy of 77.67%. Subsequently, remarkable improvements were observed by integrating attention mechanisms into the model architecture. With the attention mechanism, the model achieved a maximum AUROC score of 100% and an impressive test accuracy of 95%. This substantial enhancement in performance underscores the importance of attention mechanisms in capturing relevant features and patterns within the data, thereby facilitating more accurate classifications.

**Figure 8:**
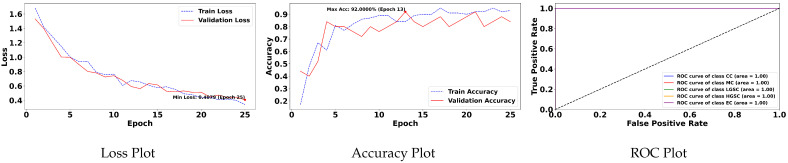
Loss, accuracy and AUROC plots for training and validation over 25 epochs for MBS = 25, with attention and top K=5 instances.

Overall, this study’s findings underscore the significance, as shown in Figure 8, of the impact of attention mechanisms on the proposed model’s performance, particularly in the context of subtype classifications. The results demonstrate the model’s ability to effectively leverage attention mechanisms to achieve superior performance metrics, thereby contributing to classification accuracy and discriminative power advancements.

### 6.2. Intra-Domain Generalization

The curated version of the original (UBC-OVARIAN) dataset inherently contains domain variations due to its multi-institutional collection protocols, including (but not limited to) featuring diverse staining protocols, scanning systems, and tissue preparation mechanisms. The intrinsic heterogeneity provides a natural testbed for evaluating domain generalization. In Table 6, we list strategically designed data splits termed as intra-domains.

Although Table 5 presents experimentation across two different splits, which implicitly supports domain generalization. But to explicitly evaluate the impact of domain generalization, we systematically designed four distinct domain splits using stratified sampling to ensure balanced class distribution across domains. Each domain was treated as a separate test set, while models were trained on the remaining three domains, simulating real-world scenarios where models encounter data from previously unseen institutions. Our framework demonstrated robust domain generalization, as evidenced by consistent performance across different data splits. As shown in Table 7, the model maintained stable performance with a maximum domain gap of only 1.3% between the best- and worst-performing domains, achieving an average cross-domain accuracy of 84.7 ± 0.6%. Furthermore, in Table 8, we reported relative differences between obtained accuracies with respect to source domains and the best reported accuracy within this study. Such $\Delta_{1}$ is the relative difference between the source domain and current domain, while $\Delta_{2}$ is introduced for the difference calculation between the best reported accuracy with regard to the target domain.

The intra-domain experiments are performed with consistent experimental settings of 16 as a batch size, 25 as an epoch size, and a bag size with ResNet50 as one of the pre-trained models. Such intra-domain experiments are recommended to be taken into consideration with other hyperparameter selections to evaluate robustness.

### 6.3. Ablation Analysis

This ablation-based experiment was designed after the successful execution of various comparative-based experiments, from which we decided to analyze the impact of various MBS with a constant feature extractor, batch size, data split ratio, and consistent epochs. Furthermore, temperature τ-based ablation is also conducted to analyze the impact of finding whether WSI-P2P is robust to temperature changes.

#### 6.3.1. Bag-Level Ablation

Table 9 documents the bag-level ablation. From experiments, it is observed that increasing the bag size (increasing instances in a bag) helps models to learn more representations and also helps with generalization, possibly influencing model performance. Specifically, a larger bag size enables models to learn diverse representations and facilitates better generalization.

#### 6.3.2. Calibration Ablation

We experiment with different values for temperature τ to assess its impact on the proposed WSI-P2P. The motivation for this calibration experiment is taken after the foundational work [65]. Many other MIL-based works have also performed this ablation to validate the proposed framework’s robustness. For this model calibration ablation, we modified the MILModel class with softmax to adjust the τ factor, which is defined as

(12) $$\mathrm{softmax}\left(z_{i}\right)=\frac{e^{z_{i}/\tau }}{\sum_{j}e^{z_{j}/\tau }}$$

In softmax’s context of temperature scaling, adjusting the temperature parameter can influence the output probabilities and be used to adjust the sharpness or softness of the output probability distribution generated by the softmax function. It involves dividing the logits (outputs before applying softmax) by a temperature parameter before using the softmax operation. The softmax operation then converts these adjusted logits into probabilities.

Table 10 presents a calibration experiment with varying values of τ, where WSI-P2P-ResNet18 shows an improvement in ACC, AUC, and F1-score when τ rises from 0.1 to 2. Performance decreases slightly above τ=2. This shows that this model architecture may benefit from a moderate temperature of around 2. Meanwhile, for WSI-P2P-ResNet50, the trend is less consistent. As τ increases from 0.1 to 0.5, the model’s performance significantly improves, suggesting that raising the temperature is beneficial. Increasing τ beyond 0.5 resulted in a decline in performance. These findings demonstrate the importance of temperature scaling in MIL tasks. The ideal temperature value depends on the feature extractor design. The higher temperature of two helps WSI-P2P-ResNet18, while 0.5 helps WSI-P2P-ResNet50. It also validates that there is no universal rule for choosing the temperature parameter, and it often requires experimentation to find the optimal value for a particular application.

### 6.4. MIL Aggregators: K-TOP Tiles Superiority

In this ablation, four aggregation methods are comparatively evaluated as discussed in the problem formulation.

**Figure 9:**
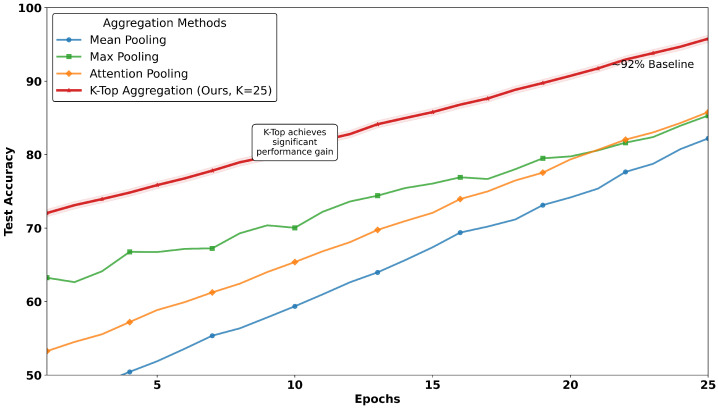
Test accuracy comparison of different validated MIL aggregation methods across an epoch size of 25. Our proposed WSI-P2P maintains a consistent performance advantage over conventional pooling methods, achieving 95% accuracy while the baseline remains under 92%.

Table 11 and Figure 9 together illustrate the comparative analysis of MIL aggregators, where the proposed work reached a peak of 95% accuracy on test data over conventional methods. The plot as depicted in Figure 9 tracks test accuracy over epochs, revealing that the method *K-TOP* achieves a consistent and significant performance advantage, while baseline methods remain below 95%, where mean pooling methods reach 81% and max pooling touches 84%. In contrast to mean and max pooling, the attention-based MIL aggregator method reaches 86%. This trend highlights the robustness of the proposed work with *K-TOP*, as it maintains its lead throughout the training process, suggesting stable convergence and effective learning dynamics. In contrast, Table 11 provides a detailed multi-metric comparison, reinforcing the findings from the image.

### 6.5. SOTA Analysis

We assessed the proposed method against several baseline methods (state-of-the-art) to validate its effectiveness. These baseline methods, established benchmarks in the field, enabled us to assess the strengths and advancements of our approach critically. The baseline, including ABMIL (“attention-based MIL”), proposed by [59], employs attention mechanisms within a MIL context to weigh instances within a bag, enhancing both performance and interpretability. This method sets a precedent for leveraging instance-level features to inform bag-level predictions, providing a robust baseline for comparison. Next, CLAM (“clustering-constrained-attention MIL”) introduced by [5], uses attention-based learning to identify sub-regions of high diagnostic value to accurately classify whole slides and instance-level clustering over the identified representative regions to constrain and refine the feature space specifically for subtype classification. A few recent works, such as DSMIL (“Dual-Stream MIL”) [66], TransMIL (“Transformer-based MIL”) [67], DFTD-MIL (“Double-tier feature distillation MIL”) [68], IBMIL (“Interventional-bag MIL”) introduced by Lin et al. [69], Lastly, MHIM-MIL [70] (“Masked Hard Instance Mining MIL”).

Table 12 illustrated the performance of the proposed method with existing SOTA methods. This table references the task category while our proposed WSI-P2P is compared with the recent work [12] presented on the same UBC-Ovarian Cancer Challenge dataset. The limitation of OCCNet [12] is that it only utilizes one-vs.-1, which means the complete WSI is treated as a single tile or patch. It is time efficient but very hard to deploy in the clinical environment and useless for robustness purposes. Medical practitioners cannot rely on subtype classification on a single tile, while our methodology intuitively employs 50 tiles and presents a competitive performance, and further refinement is needed. The single-tile selection approach shows an F1-Score of 93.67% while it achieved 93% for balanced accuracy. In comparison to OCCNet, WSI-P2P yields a maximum score of 95.89% AUROC and a test accuracy of 77.67% without attention; further, 100% AUROC and a test accuracy of 95% are recorded with the attention mechanism, demonstrating excellent performance between subtype classifications. A comparative analysis of WSI-P2P employing SOTA methods with diverse datasets is suggested, and it would further benefit computational pathology and justify the applicability of the proposed method.

After conducting a comparison with SOTA MIL techniques, we conducted a detailed investigation on recent works on UBC-Othe UBC-OCEANset and prepared a comparative literature table as recorded in Table A2, which provides comprehensive information with respect to key aspects and emphasizes key contributions along with limitations. Finally, we compared the proposed *K-TOP* MIL aggregator on the test dataset with existing MIL aggregators over 25 epochs for K=25, which outperformed our own K=5. The results are summarized in Figure 9.

### 6.6. Model Inference Analysis

The average time from various experiments based on 25 epochs was recorded as 38 min or 0.63 h for One-vs.-fifty tiles/WSI for our proposed approach, while for the rest of the compared methods, the time varies from 1.12 h to 2.48 h per 25 epochs with identical experimental conditions; this represents a 2.8× to 4.9× superiority in terms of speed, attributed to the adopted key innovations.

We also evaluated computational efficiency by measuring the relative model inference speed in terms of times × as reported in Table 11. Attention Pooling (normalized to 1.0×) is regarded as baseline. Ours *K-TOP* = 5 demonstrates a significant 2.3× speedup over standard attention mechanisms while maintaining a competitive performance, achieving an optimal balance between computational efficiency and classification accuracy. The *K-TOP* = 25, while slightly slower at 1.5× speedup in comparison to the fastest reported Max Pooling, reported the highest accuracy at 95.72%, illustrating the flexibility of our method in trading off between computational demands and performance requirements.

## 7. Limitations and Future Work

The success of *K-TOP* aggregation attributed to its ability to selectively focus on the most informative instances within a bag, effectively filtering out noise and irrelevant data. Unlike mean pooling, which dilutes signals by average outing all instances, or max pooling, which risks overemphasizing outliers, the proposed method presents an optimal balance by aggregating top *K* instances. This approach is especially advantageous in domains like computational pathology, where WSI contains vast amounts of data, but only a small subset of patches are diagnostically relevant. The method’s consistent high accuracy and AUROC across a comprehensive set of experiments suggest strong potential for clinical deployment.

However, we acknowledge that the current study has several limitations. Firstly, the framework’s validation is primarily conducted on a single dataset, limiting assessments of its generalization across diverse population demographics and institutional protocols. Secondly, the absence of long-term clinical validation prevents definitive conclusions about real-world utility. Lastly, while *K-TOP* aggregation demonstrates strong performance, further fine-tuning and optimization could enhance its robustness across varying tumor densities and morphological patterns.

Building upon the foundation, we outline several perspectives for future work:

- Adoption of Datasets: Additional comprehensive evaluation of multiple datasets with diverse patient populations and staining protocols is recommended to assess true generalization. To achieve this, exploration of public and private datasets is suggested.
- Domain Generalization: Future works may investigate *K-TOP* aggregation’s effectiveness for other cancer types and histopathological tasks beyond ovarian cancer classification.
- Architectural Advancements: Exploration of transformer-based architectures for enhanced feature representation, including vision transformers (ViTs) for patch-level analysis by integrating *K-TOP* aggregation for improved long-range dependency modeling. Further, multimodal data (genomic, clinical, and radiomic features) could further enhance diagnostic accuracy and clinical relevance. Development of advanced explainability techniques, including quantitative validation of attention maps against pathologist annotations and integration with clinical decision support systems.
- Interpretability Enhancement: Lastly, we highly recommend implementation of rigorous quantitative assessment for model explainability (e.g., saliency map), including pathologist-in-the-loop validation of attention mechanisms and statistical correlation analysis between model focus regions and clinically relevant histopathological features.

In summary, the WSI-P2P framework represents significant progress toward developing efficient, scalable, and generalizable computational pathology tools. This study not only contributes to the evolution of digital pathology but also establishes a foundation for future research in domain-generalized whole-slide image analysis.

## 8. Conclusions

This study introduced WSI-P2P (Whole-Slide Imaging–Patch to Prediction), a novel framework for domain-generalized ovarian cancer classification that integrates downscaled sampling, multiple instance learning, and transfer learning. The framework addresses key challenges in computational pathology by providing a robust performance across heterogeneous data sources while maintaining computational efficiency, offering a solution to the limitations in conventional H&E-based diagnosis where interobserver variability remains high and specialized expertise is limited.

The integration of transfer learning with a MIL framework allowed WSI-P2P to learn and make predictions adaptively on data characterized by inherent variability and complexity. Downscaled patch sampling has further shown computational efficiency, allowing for vast WSI datasets to the processed without compromising the integrity of the histological features critical for accurate classification. Furthermore, the impact of attention mechanisms with *K-TOP* aggregation showed significant progress in the proposed model’s performance, particularly in the context of subtype classifications. Lastly, the framework demonstrated exceptional domain generalization capabilities, validating the robustness of *K-TOP* aggregation for histology images and emphasizing WSI-P2P as a promising tool for reliable ovarian cancer subtyping across diverse clinical settings.

## Figures and Tables

**Table 1:** The curated dataset statistics (ICWC—initial class-wise WSI count) and (PCWC—preprocessed class-wise WSI count). The tile count is a multiple of the bag size of 50 (e.g., 41×50=2050).

Abbreviation	Class	ICWC	Nature	PCWC	Nature	Tile Count
Mucinous Carcinoma	MC	46	One-vs.-1	41	One-vs.-50	2050
Low-Grade Serous Carcinoma	LGSC	47	One-vs.-1	42	One-vs.-50	2100
Clear Cell Carcinoma	CC	99	One-vs.-1	94	One-vs.-50	4700
Endometrioid Carcinoma	EC	124	One-vs.-1	119	One-vs.-50	5950
High-Grade Serous Carcinoma	HGSC	222	One-vs.-1	217	One-vs.-50	10,850
Total	-	538	-	513	-	25,650

**Table 2:** Summary of dataset histology image characteristics: width and height Z-score distribution for possible outlier detection (aka OOD: out of distribution).

Image_Id	Label	Image_Width	Image_Height	Is_Tma	Width_Z Score	Height_Z Score
4	HGSC	23,785	20,008	False	1.25116	0.903238
66	LGSC	48,871	48,195	False	0.000572155	1.71567
91	HGSC	3388	3388	True	2.26893	2.44743
281	LGSC	42,309	15,545	False	0.326857	1.3179
286	EC	37,204	30,020	False	0.581585	0.0269945

**Table 3:** Experimental parameters with rationale.

Parameter	Rationale	Specific Value
Input dimensions	WSI representation: batch size × bags × tiles × channels × spatial dimensions	(N, B, T, C, H, W) = e.g., (8, 1, 25, 3, 224, 224)
Number of Classes	Five ovarian cancer subtypes following WHO classification standards	5 classes: [“CC”, “MC”, “LGSC”, “HGSC”, “EC”]
Data Augmentation	Histology-specific transformations to improve domain generalization and prevent overfitting	Random rotation, flipping, color jitter, random crop, Gaussian blur
Learning Rate	Balanced convergence speed and stability with OneCycleLR scheduling for optimal performance	$2\times 10^{-4}$
Training Epochs	Sufficient for convergence while preventing overfitting, with early stopping patience	25 epochs
Batch Size	Memory-efficient processing with gradient accumulation for effective batch size	[A series of experimentats with 4, 8, and 16]
MIL Aggregation	Comparative analysis of aggregation strategies with K-TOP for computational efficiency	[Min, Max, Mean, Attention, K-TOP (K=5)]
Data Splitting	Overall, various data splits were evaluated. For domain generalization evaluation through cross-domain validation strategy	4-domain cross-validation (80%, 20%)
Feature Extractor	Pre-trained backbone with progressive unfreezing for transfer learning	ResNet-18 and ResNet-50 (ImageNet pre-trained)
Optimizer	Adaptive learning with weight decay for regularization	AdamW ($\beta_{1}=0.9$, $\beta_{2}=0.999$, weight decay = $1\times 10^{-4}$)
Loss Function	Objective function for training	CrossEntropyLOss
Tiles per Bag	Computational efficiency while maintaining diagnostic information	Max available 50 tiles per WSI (while, K=5 for aggregation)
Image Resolution	Standard input size for pre-trained models with preserved histological features	224×224 pixels

**Table 4:** Metrics for WSI-P2P with a LinearNN Classifier (frozen feature extractor + linear layer, no attention or fine-tuning), tested across batch sizes, data splits, and MIL Bag Sizes (MBS) [Tr is short for Training, Vl is used for Validation, and Ts is defined for Testing Instances] shows the best-recorded metrics. Without attention (base model).

MBS	Data Split (Tr,Vl,Ts)	Feature Extractor	Batch Size	Accuracy	Precision	Recall	F1-Score	BlAcc	Ch. Kappa	AUROC	AUPRC
10	(128,128,257)	WSI-P2P-ResNet18	16	0.6148	0.6248	0.6148	0.6125	0.4915	0.4566	0.8414	0.5631
			32	0.6225	0.6164	0.6225	0.6076	0.4893	0.4528	0.8351	0.5966
			64	0.6693	0.6922	0.6693	0.6452	0.5444	0.5319	0.8436	0.6393
	(328,82,103)		16	**0.7767**	0.7957	0.7767	**0.7682**	0.6862	0.6727	0.9387	0.8284
			32	0.701	0.7026	0.7087	0.7045	0.6777	0.5828	0.8887	0.7452
			64	0.6796	0.6993	0.6796	0.6633	0.5535	0.5176	0.8216	0.6622
	(128,128,257)	WSI-P2P-ResNet50	4	0.6187	0.6448	0.6187	0.6194	0.5624	0.4702	0.8543	0.6189
			16	0.7471	7442	0.7471	0.7366	0.6848	0.6332	0.8965	0.7545
	(328,82,103)		4	0.7184	0.7130	0.7184	0.7081	0.6004	0.5863	0.913	0.7325
			16	0.7573	0.7532	0.7573	0.7522	0.6912	0.6487	0.9220	0.788
25	(128,128,257)	WSI-P2P-ResNet50	4	0.7082	0.7162	0.7082	0.6977	0.6226	0.5871	0.8672	0.681
			8	0.6809	0.6904	0.6809	0.6684	0.5938	0.5339	0.8946	0.7
	(328,82,103)		4	0.7184	0.7079	0.7184	0.7074	0.6683	0.5831	0.9135	0.7754
			8	0.7573	0.7647	0.7573	0.7600	**0.7153**	0.6639	0.9193	0.7947
50	(128,128,257)	WSI-P2P-ResNet18	4	0.6031	0.5853	0.6031	0.5908	0.5073	0.4379	0.8338	0.5985
	(328,82,103)			0.7282	0.7627	0.7282	0.7056	0.6327	0.6215	**0.9589**	0.8669
	(128,128,257)	WSI-P2P-ResNet50		0.7393	0.7438	0.7393	0.7275	0.6717	0.6263	0.917	0.7776
	(328,82,103)			0.7695	0.7521	0.7695	0.7358	0.6818	0.6536	0.920	0.7850

**Table 5:** Performance metrics of various experiment configurations during training of WSI-P2P. The listed records are sorted by validation accuracy, presenting the top 12 results for emphasis on the proposed *K-TOP* selective approach, with the maximum value highlighted as **bold**, while the average among results is underlined and the worst results are shown in *italics*. Additionally, the loss, Accuracy, and AUROC plot over epochs are in Figure 5 for the configuration with the highest score. The configurations are specified as D1 = (128,128,257), while D2 = (328,82,103). With attention only.

Exp Nature	Max Val Accuracy	Min Val Loss	Max Train Accuracy	Min Train Loss
MBS25-ResNet50-Batch8-D2	**0.8414**	**0.4351**	**0.9054**	**0.3041**
MBS25-ResNet50-Batch4-D2	0.8292	0.7437	0.9054	0.3074
MBS10-ResNet50-Batch4-D2	0.8171	0.7164	0.8871	0.3175
MBS10-ResNet50-Batch16-D2	0.7926	0.7128	0.9329	0.2287
MBS10-ResNet18-Batch16-D2	0.7682	0.7301	0.8750	0.3756
MBS50-ResNet18-Batch4-D2	0.7682	0.6343	0.8231	0.4831
MBS25-ResNet50-Batch4-D1	0.7265	0.8858	0.9062	0.2856
MBS10-ResNet50-Batch16-D1	0.7265	0.7521	0.9609	0.2429
MBS10-ResNet18-Batch32-D2	0.7073	0.8732	0.8689	0.4271
MBS10-ResNet18-Batch64-D2	0.7000	0.9001	0.8292	0.5122
MBS10-ResNet18-Batch32-D1	0.6718	0.8603	0.9141	0.3718
MBS50-ResNet50-Batch4-D1	*0.6406*	*1.0441*	*0.9843*	*0.1222*

**Table 6:** Data splits for the intra-domain generalization experiment. D is short for domain, followed by a number as a suffix.

Domain	Training WSI	Testing WSI	Total WSI
D1	102	25	127
D2	102	25	127
D3	102	25	127
D4	104	28	132
Overall	410	103	513

**Table 7:** Intra-domain generalization performance. Domain gap (%) is a relative difference between accuracies.

Domain	Accuracy	BlAcc	AUROC	F1-Score	Domain Gap (%)
D1	85.2	72.7	94.8	83.9	-
D2	83.9	71.4	93.7	82.1	1.3
D3	84.6	72.2	94.2	83.0	0.7
D4	85.1	72.5	94.5	83.7	0.5
Average	84.7 ± 0.6	72.2 ± 0.5	94.3 ± 1.25	83.2 ± 0.8	0.8 ± 0.4

**Table 8:** Relative differences in accuracy across intra-domains. $\Delta_{1}$ is from the source (85.2%), while $\Delta_{2}$ is from the best (95.72%). Here, we report 0–9% as indicative of high consistency, while above 9% is regarded as medium consistency.

Domain	Accuracy	$\Delta_{1}$	$\Delta_{2}$	Domain Consistency (in Pair)
D1	85.2	-	10.52	(-, medium)
D2	83.9	1.3	11.82	(high, medium)
D3	84.6	0.7	11.12	(high, medium)
D4	85.1	0.5	10.62	(high, medium)

**Table 9:** Ablation analysis of MIL bag size (MBS) across one-vs.-5 to one-vs.-50 WSI patches with a consistent feature extractor, batch size, epochs, and data split.

	WSI-P2P-ResNet18			WSI-P2P-ResNet50		
**MBS**	**Accuracy**	**AUROC**	**F1-Score**	**Accuracy**	**AUROC**	**F1-Score**
5	0.5370	0.8003	0.5465	0.4864	0.8014	0.5266
10	0.5486	0.8509	0.5430	0.6265	0.8707	0.6234
15	0.5720	0.8270	0.5317	0.6109	0.8817	0.6203
25	0.6693	0.9097	0.6539	0.6381	0.8884	0.6109
50	0.8390	0.9528	0.7621	0.8821	0.9623	0.7726

**Table 10:** WSI-P2P calibration with temperature τ with a constant experimental setting, such as MBS = 15, feature extractor (ResNet18 and ResNet50), batch size (16), data split ratio (128,128,257), and consistent epochs (25). Further, experimentation on τ can be explored for other variants of MBS.

Feature Extractor	τ	Accuracy	AUROC	F1-Score
WSI-P2P-ResNet18	0.1	0.4747	0.6750	0.4143
	0.3	0.5564	0.7568	0.5156
	0.5	0.5759	0.7200	0.5304
	1	0.5564	0.7565	0.5073
	2	0.5798	0.7822	0.5167
	5	0.5292	0.7611	0.4476
WSI-P2P-ResNet50	0.1	0.5681	0.7216	0.5308
	0.3	0.5759	0.7346	0.5321
	0.5	0.6342	0.7903	0.5819
	1	0.6019	0.7866	0.5551
	2	0.6264	0.7947	0.5877
	5	0.6108	0.8031	0.5486

**Table 11:** MIL aggregators’ comparative analysis with MBS = 50, with complete curated dataset samples with a split of (80%, 20%). IT is used instead of inference time, where we reported time in terms of computational speed with respect to method; relative discussion is discussed in Section 6.6.

Aggregator	Accuracy	AUROC	F1-Score	IT
Mean Pooling	0.8130 ± 1.2	0.8901 ± 0.02	0.8101 ± 0.03	≥2.8×
Max Pooling	0.8460 ± 0.9	0.9100 ± 0.01	0.8301 ± 0.02	≥3.2×
Attention Pooling	0.8650 ± 0.8	0.9300 ± 0.01	0.8500 ± 0.02	≈1.0×
***K-TOP*** **(Ours, K=5)**	**0.9200 ± 0.01**	**0.9900 ± 0.01**	**0.8624 ± 0.01**	2.3×
***K-TOP*** **(Ours, K=25)**	**0.9572 ± 0.6**	**0.9900 ± 0.01**	**0.8700 ± 0.01**	1.5×

**Table 12:** Comparison of Various SOTA MIL Methods with regard to WSI-P2P. IT is short for Inference Time (h/25 epoch).

Methods	WSI-P2P METRICS				IT
	**Accuracy**	**AUROC**	**F1-Score**	**BlAcc**	
CLAM [5]	85.14 ± 0.85	89.70 ± 0.76	82.10 ± 0.63	59.00 ± 0.09	1.15
LiteMIL [33]	92.40 ± 0.01	98.7 ± 0.02	89.2 ± 0.01	63.37 ± 0.29	1.12
AB-MIL [59]	90.06 ± 0.72	94.54 ± 0.30	87.83 ± 0.83	63.86 ± 0.58	1.24
DSMIL [66]	90.17 ± 0.02	94.57 ± 0.40	87.65 ± 0.18	48.84 ± 0.19	2.48
TransMIL [67]	81.22 ± 0.32	85.51 ± 0.13	79.10 ± 0.33	58.64 ± 0.28	1.35
DTFD-MIL [68]	90.22 ± 0.36	95.15 ± 0.14	88.4 ± 0.11	61.21 ± 0.13	1.80
IBMIL [69]	75.23 ± 0.41	82.80 ± 0.03	74.35 ± 0.89	55.62 ± 0.38	1.37
MHIM-MIL [70]	89.16 ± 0.01	90.14 ± 0.02	81.94 ± 0.70	61.14 ± 0.13	1.14
FOCUS (BaseMIL) [71]	91.81 ± 0.19	91.10 ± 0.03	65.6 ± 0.09	70.4 ± 0.08	1.2
WSI-P2P	**95.72%**	**100%**	**92%**	**76.82%**	0.63

## Data Availability

The original version of the dataset and relevant code files will be available in the GitHub repository (https://github.com/drahmedawais/WSI-P2P), while a curated subset of the dataset will be available on reasonable request.

## Appendix A

### Appendix A.1. Integration of Human-in-the-Loop

While the model-generated heatmaps, as shown in Figure 7, provide compelling visuals of the areas of focus, it’s crucial to quantitatively align these regions with diagnostically relevant tissues to establish clinical trust and utility. Although the heatmaps indicate clinically relevant areas through high activation, a thorough quantitative evaluation is necessary to validate these findings. Integrating a human-in-the-loop approach could involve collaborating with pathologists to assess and verify highlighted regions, ensuring that the model’s predictions align with expert clinical judgment. This validation process could enhance the model’s explainability. In one of the review study by Paracchini et al. [14], presented conclusive remarks on how AI explainability can effectively bridge algorithmic and clinical domains, emphasizing the significance of collaborative evaluation in advancing AI application in medical contexts.

### Appendix A.2. Translational AI Frameworks in Pathology

The translational AI framework aims to bridge the gap between research and real-world applications by facilitating the practical development of AI in clinical pathology.

Restaino et al. [13] provide a comprehensive examination of AI integration across gynecological oncology, emphasizing that successful clinical translation requires addressing workflow integration, regulatory considerations, etc. The study [14] demonstrated the importance of standardized validation and explainability for surgical AI systems, principles that directly translate to pathology applications. Their systematic review underscores that clinician trust and adoption heavily depend on model interpretability and consistent performance across varied institutional settings. Furthermore, advancing human-in-the-loop collaboration would enhance possibilities of real-time execution and deployments [15]. Additionally, investigating comparative performance between AI systems and medical professionals would help in developing output-centric solutions, ultimately enhancing patient outcomes.

As demonstrated by the recent advancements in computational pathology [58], to fully cope with practical deployment, the next steps involve several key strategies:

- Data standardization and annotation
- Interdisciplinary collaboration
- Regulatory and Ethical Considerations
- Human-in-the-Loop
- Implementation and continuous learning

These translational frameworks collectively demonstrate the next-generation medical AI must address deployment challenges with the same rigor as technical performance metrics. The presented WSI-P2P framework contributes to this specific area by specifically targeting key deployments in computational pathology, where the focus remains on reducing computational power and increasing efficiency for practical clinical use, enhancing robust domain generalization that potentially facilitates pathologists, making it a promising application of deep learning for histology images.

### Appendix A.3. Appendix Tables

In this appendix we present a list of key terms used in this study with their full abbreviations as recorded in Table A1, while in Table A2, we recorded detailed analysis of recent works on the UBC-OCEAN dataset with respect to their computational requirements and performance.

**Table A1:** A table of terms with their full abbreviations used within the study.

Term	Full Abbreviation
AI	Artificial Intelligence
AUROC	Area Under Receiver Operating Characteristic
BlAcc	Balance Accuracy
CC	Clear Cell Carcinoma
CP	Computational Pathology
EC	Endometrioid Carcinoma
EGSO	European Society of Gynecological Oncology
HGSC	High-Grade Serous Carcinoma
IT	Inference Time
OOD	Out-of-Distribution
LGSC	Low-Grade Serous Carcinoma
MC	Mucinous Carcinoma
MIL	Multiple Instance Learning
MBS	MIL Bag Size
WHO	World Health Organization
WSI	Whole Slide Image
WSI-P2P	Whole Slide Imaging-Patch to Prediction
UBC-OCEAN	“UBC Ovarian Cancer subtypE clAssification and outlier detectioN”

Table A2 presents a detailed analysis of computational requirements and performance metrics across recent approaches. In summary, our approach processes only 50 tiles per WSI compared to 200 in [72], while achieving significantly higher performance, with memory reduction recorded at 4×. While some methods like [12] reported high balanced accuracy, they rely on single-instance processing, which lacks clinical relevance. Unlike approaches focusing solely on classification performance or feature extraction, our proposed WSI-P2P specifically addresses the computational challenges of WSI processing through down-scaling and selective *K-TOP* aggregation. Our approach also demonstrated significant improvements in inference time, outperforming methods that require higher computational resources.

**Table A2:** Computational Advantage Metrics of WSI-P2P w.r.t. SOTA for UBC-OCEAN Challenge Dataset. N/A is used where information is not available, while N/C is used where particular information is not clear from the methodology. Tiles/WSI (Here One-vs.-N (1,50,200) Means 1 Patch of 1 WSI, 50 Patches of 1 WSI, and 200 Patches of 1 WSI, Respectively). * We estimated their time and memory to the best of our understanding.

Ref	Key Discussion (Methodology)	Tiles/WSI	Memory/WSI	Inference Time	Result Metrics	Limitation
[72]	ViT-Ensemble: The UBC-OCEAN winner author’s used ensemble of Chowder models on top of Phikon tile embeddings. With over 10,000 patches for each WSI, they selected 200 patches to reduce computational complexities.	One-vs.-200	4× of ours *	4× to 8× of ours *	66% BlAcc	The scientific work is not yet proposed, so can’t say anything about practicality.
[12]	OCCNet: Ensemble Attention Mechanism (EAM) based solution based on single patch of each WSI, main objective were handling unbalanced feature representation during model training.	One-vs.-1	N/A	N/A	93% BlAcc	Study solely relies on single instance of WSI, which is computationally effective but result generated by proposed solution is not clinically relevant.
[41]	This is competition summary and comparative based study, hence no particular methodology discussed	N/A	N/A	N/A	Various balanced accuracies were reported as 61% to 68% on public dataset while 58% to 66% on private dataset	Particularly focuses on subtype classification rather than reducing computational burden of WSI for overall processing.
[73]	Msa-MIL-Net: An end-to-end multi-scale aware MIL method is proposed, where various magnification of same WSIs are generated consequently MIL bags are trained on proposed model.	One-vs.-150	N/A	N/A	An AUC of 95% reported while Accuracy is reported as 93%	While proposed method achieved competitive results, their focus is on multi-scale based classification, while the study is not indenting to reduce computational complexities.
[31]	Top-OC: The authors proposed topological deep learning framework for Ovarian based on variants of features	N/A	N/A	N/A	Study reported 66.13% BlAcc, 72.45% Accuracy and 91.74% AUC.	While proposed method achieved competitive results, their focus is on topological data analysis while the study is not indenting to reduce computational complexities, instead they validated various magnification level.
[8]	The proposed study utilized deep fine-KNN classification.	One-vs.-1	N/A	N/A	Average AUC of 85.4%	While the proposed strategy is effective and yields strong results in subtype classification, the technique solely depends on individual WSI instead of a bag of instances. Additionally, authors relied on a single feature extractor, which is a potential bias.
[74]	The study presented detailed investigation on Phikon feature extractor, where they utilized ABMIL techniques on different cancer sites.	N/C	N/A	N/A	87.4% AUC	The study primarily relies on comparing feature extractors itself rather than histology comparisons
[71]	The study proposed a comprehensive framework for knowledge-enhanced adaptive visual compression for WSI classification, where they compared several methods, including MIL, referred to as BaseMIL.	N/A	N/A	N/A	70.4% BlAcc	The study primarily compares various variants of few-shot, rather than presenting a competitive framework for histology classification
WSI-P2P	The framework presents an innovative approach for integrating down-scaling and *K-TOP* multiple instance learning (MIL) aggregator, specifically targeting the classification of ovarian cancer histology. Further, it enhances computational efficiency by optimizing MIL aggregation through selective instance processing. Additionally, WSI-P2P leverages transfer learning for robust domain generalization. It outperforms existing works in terms of performance.	One-vs.-50	3.2 GB	0.63 h/25 epoch	95% Accuracy and 76.8% BlAcc	We achieved moderate balanced accuracy. The approach requires tile pre-filtering and down-scaling, which is huge work.
